# Rivaroxaban as an Antithrombotic Agent in a Patient With ST-Segment Elevation Myocardial Infarction and Left Ventricular Thrombus: A Case Report

**DOI:** 10.1177/2324709617697991

**Published:** 2017-03-23

**Authors:** Rajeev Seecheran, Valmiki Seecheran, Sangeeta Persad, Naveen Anand Seecheran

**Affiliations:** 1 The University of the West Indies, St Augustine, Trinidad and Tobago; 2 North West Regional Health Authority, Port of Spain, Trinidad and Tobago; 3 North Central Regional Health Authority, Chaguanas, Trinidad and Tobago

**Keywords:** STE-ACS, left ventricular thrombus, rivaroxaban, ticagrelor

## Abstract

The incidence of left ventricular (LV) thrombi in the setting of an anterior myocardial infarction has declined significantly since the advent of primary percutaneous coronary intervention coupled with contemporary antithrombotic strategies in ST-segment elevation myocardial infarctions (STE-ACS). Despite oral anticoagulation with the currently accepted, standard-of-care vitamin K antagonist, warfarin, major bleeding complications still arise. Rivaroxaban is a novel, direct oral factor X anticoagulant that has several advantageous properties, which can attenuate bleeding risk. We present a case in which a patient successfully underwent a 3-month course of rivaroxaban in addition to his dual antiplatelet regimen of aspirin and ticagrelor for his STE-ACS and LV thrombus with resultant complete dissolution.

## Introduction

The incidence of left ventricular (LV) thrombi in the setting of an anterior myocardial infarction has declined significantly since the advent of primary percutaneous coronary intervention coupled with contemporary antithrombotic strategies in ST-segment elevation myocardial infarctions (STE-ACS).^1^ Despite these milestones, apical LV thrombi are still identified in approximately 5% of anterior STE-ACS.^1^ Presently, oral anticoagulation with a vitamin K antagonist (VKA), for example, warfarin, is considered the standard of care per the European Society of Cardiology guidelines.^2^ Additionally, similar recommendations have been issued by the American College of Cardiology/American Heart Association (ACC/AHA) guidelines, which target an international normalized ratio of 2.0 to 3.0, for at least 3 months (Class I, Level of Evidence B) and, perhaps, indefinitely in patients without an increased risk of bleeding (Class I, Level of Evidence C).^3^ Warfarin, however, has several liabilities such as an initial phase of hypercoagulability^4^ and variable anticoagulation with treatment being associated with both sub- and supratherapeutic effects.

Recently, novel direct oral anticoagulants (DOACs) have emerged as an alternative to VKAs for several indications and are Food and Drug Administration–approved for pulmonary embolism (PE) and nonvalvular atrial fibrillation (NVAF).^5^ Advantageous characteristics of DOACs include a favorable safety profile, more rapid onset of effect, more consistent anticoagulation without the need for regular monitoring, and typically fewer drug interactions. There currently exists a paucity of data with respect to the efficacy and safety of DOACs in treating LV thrombi associated with STE-ACS status post DES (drug-eluting stent) on DAPT (dual antiplatelet therapy), specifically in combination with ticagrelor. Prior studies have assessed the use of DOACs for intracardiac thrombi dissolution in several settings including LV outflow tract obstruction, LV apical dyskinesia, tachycardia-mediated cardiomyopathy, and left atrial appendage.

## Case Report

**Figure 1.**
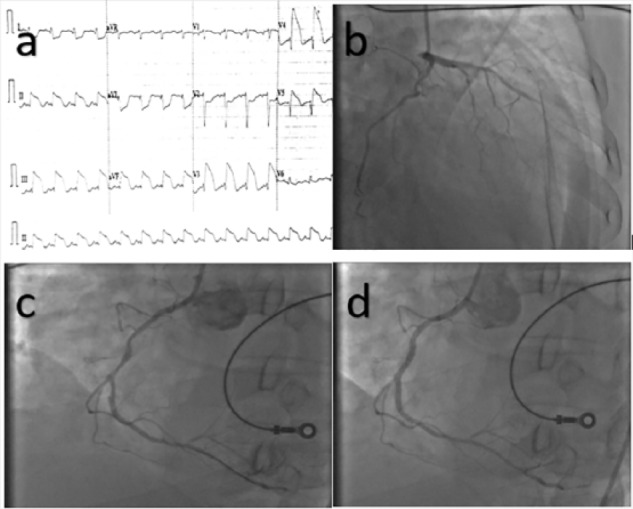
(a) Diffuse ST-segment elevations in both anterior and inferior leads, suggestive of global ischemia. (b) A chronic total occlusion of the mid left anterior descending artery with right to left collaterals. (c) The culprit lesion in the mid right coronary artery with high thrombus burden at site of plaque rupture. (d) Successful implantation of the drug-eluting stent with a good angiographic result.

A 53-year-old South Asian male with no prior medical history presented with an anterior ST-segment elevation-ACS and new-onset diabetic ketoacidosis in cardiogenic shock. A 12-lead electrocardiogram revealed ST elevation in anterior and inferior leads with reciprocal ST depression in the lateral and septal leads (see Figure 1a). Emergent coronary angiography revealed a chronic total occlusion in the mid left anterior descending coronary artery with bridging collaterals and a ruptured, ulcerated plaque with TIMI grade 5 thrombus in the mid right coronary artery (RCA), the suspected culprit lesion (ACC/AHA Type C; see Figure 1b and c, respectively). The patient’s SYNTAX score II was 39. Percutaneous coronary intervention (PCI) was performed via manual aspiration thrombectomy, and subsequently, a 2.5 × 28 mm Boston Scientific Promus Premier (Marlborough, MA) DES was successfully implanted into the culprit vessel (RCA) with a good angiographic result and no complications (see Figure 1d).

**Figure 2.**
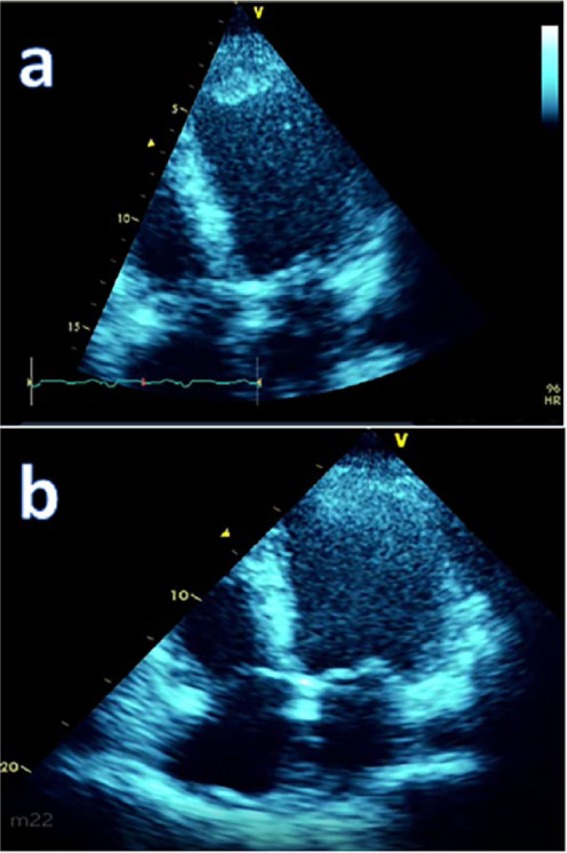
(a) The apical 25 mm × 15 mm left ventricular thrombus. (b) Complete dissolution of the preexisting apical left ventricular thrombus after adopting the antithrombotic strategy.

Prior to PCI, the patient was treated with an insulin infusion and an antithrombotic strategy that consisted of aspirin 81 mg daily, ticagrelor 180 mg load, followed by maintenance dose of 90 mg twice daily and a therapeutic dose of subcutaneous enoxaparin (80 mg). Post-PCI 2D-transthoracic echocardiography (2D-TTE) revealed a large apical thrombus with an LV ejection fraction of approximately 25% (see Figure 2a). The calculated HAS-BLED score was 1, which translated to a 3.4% risk of a bleeding event per year. The patient was maintained on aspirin and ticagrelor for his STE-ACS status post DES PCI. Enoxaparin was discontinued and rivaroxaban was initiated in view of his apical thrombus and extensive thrombus burden at the site of plaque rupture. He was safely discharged on optimal medical therapy comprising a beta-blocker, an angiotensin-converting enzyme inhibitor, an aldosterone receptor antagonist, and insulin therapy after a 7-day hospitalization. At 3 months, a 2D-TTE revealed complete interval dissolution of the apical thrombus; however, apical dyskinesia persisted with aneurysmal changes and a substantial improvement in ejection fraction to 37% (see Figure 2b).

Based on pharmacodynamics, pharmacokinetics, and the patient’s normal renal function, we opted for a dosage of 10 mg twice daily for 12 weeks with cessation after echocardiographic evidence of dissolution. We used a twice-daily regimen, as the majority of his optimal medical therapy were also administered twice daily and, thus, may improve his medication adherence. This permitted us to avoid traditional “triple therapy” for a protracted period. We would like to underscore that this dose was a modification of the EINSTEIN-PE treatment strategy, as the patient was exposed to imminent thromboembolic complications from the precarious LV thrombus and, overall, low risk for major bleeding.

## Discussion

Rivaroxaban is a highly selective direct factor Xa inhibitor with oral bioavailability and rapid onset of action. It has predictable pharmacokinetics across a wide spectrum of patients (age, gender, weight, race) and has a flat dose response across an 8-fold dose range (5-40 mg).^6^ It is currently approved for treatment of PE and NVAF.^7^ This case report presents a strategy using rivaroxaban along with dual antiplatelet therapy with ticagrelor to achieve both thrombus dissolution and stent patency. This regimen was selected considering his LV thrombus, extensive thrombus burden in the setting of severely uncontrolled diabetes with a glycosylated hemoglobin A1c of ~14%, and a relatively low risk of a major bleeding event.

Safety and efficacy are of paramount importance when considering a patient’s ACS regimen. Obtaining the “sweet-spot” between a drug’s optimal anti-ischemic effect and its risk of a bleeding complication may be difficult to achieve depending on the therapeutic window and patient-specific concerns. Some important aspects that should be considered include timing of the dose, duration of infusion, drug compatibility, errors in estimating a patient’s weight and/or age, failure to adjust the dosage based on renal function, and errors in drug preparation. Ideally, an individualized antithrombotic regimen in these high-risk patients should be selected. As aforementioned, diabetes is associated with increased platelet reactivity via several mechanistic effects,^8^ and studies have demonstrated that thrombin levels stay persistently elevated for months after an ACS event,^9^ hence our rationale for adopting this strategy. DOACs, such as rivaroxaban, are a viable alternative in the management of patients with PE as demonstrated in the EINSTEIN-PE trial,^10^ and as such, we selected it as our preferred anticoagulant for the LV thrombus. We acknowledge that this regimen may incur potentially devastating complications; however, after a considered view of his high-risk profile, we decided to select a regimen that would be consistent and effective.

Currently, there are no randomized trials or guideline recommendations considering the use of NOACs (novel oral anticoagulants) in combination with antiplatelet agents for patients with ACS and LV thrombi; however, there are several ongoing and recently concluded studies evaluating similar combinations of antiplatelet and anticoagulant regimens, albeit in the setting of atrial fibrillation. PIONEER AF-PCI is a recently concluded randomized comparison of VKA against NOACs therapy in patients with NVAF receiving antiplatelet therapy after PCI to assess the relative risks of bleeding complications.^11^ This study demonstrated that in participants with atrial fibrillation undergoing PCI with placement of stents, the administration of either low-dose rivaroxaban plus a P2Y12 inhibitor for 12 months (group 1) or very-low-dose rivaroxaban plus DAPT for 1, 6, or 12 months (group 2) was associated with a lower rate of clinically significant bleeding than was standard therapy with a vitamin K antagonist plus DAPT for 1, 6, or 12 months (group 3; 16.8% in group 1, 18.0% in group 2, and 26.7% in group 3).^12^ The rates of death from cardiovascular causes, myocardial infarction, or stroke were similar in the 3 groups (6.5% in group 1, 5.6% in group 2, and 6.0% in group 3)^12^; however, the rates were not statistically significant.

Prior to the PIONEER AF-PCI study, in 2012, this novel “triple therapy” synergistic strategy was assessed in the ATLAS ACS 2-TIMI 51 trial, which demonstrated than in patients with a recent ACS, rivaroxaban reduced the risk of the composite endpoint of death from cardiovascular causes, myocardial infarction, or stroke at the expense of increased risk of major bleeding and intracranial hemorrhage but not the risk of fatal bleeding.^13^ The twice-daily 2.5-mg dose of rivaroxaban reduced the rates of death from cardiovascular causes (2.7% vs 4.1%, *P* = .002) while resulting in fewer fatal bleeding events than the twice-daily 5-mg dose (0.1% vs 0.4%, *P* = .04). There is also the ongoing RT-AF trial that will provide data primarily regarding the safety of dual therapy with rivaroxaban and ticagrelor over the traditional triple therapy in patients with AF undergoing PCI at 12 months.^12^

These 2 landmark studies suggest that the addition of NOAC to DAPT may be a safer antithrombotic strategy to warfarin and DAPT as demonstrated by less bleeding with near-equivalent primary outcomes. The ongoing phase 2 trial, GEMINI-ACS-1,^14^ is comparing the safety of rivaroxaban versus aspirin in addition to either clopidogrel or ticagrelor in ACS; results will further guide appropriate post–myocardial infarction anticoagulation and antiplatelet therapies for patients who require both.

## Conclusion

Short-term rivaroxaban was effective as a component of “triple therapy” with aspirin and ticagrelor for LV thrombus dissolution. Randomized controlled trials are required to confirm these promising results and to ascertain the optimal dosage of DOACs when associated with DAPT, specifically including ticagrelor. This regimen may be a clinically effective and safe strategy in patients who are considerably high risk for thromboembolic events but are relatively low risk for bleeding complications.
